# Development of a mobbing short scale in the Gutenberg Health Study

**DOI:** 10.1007/s00420-015-1058-6

**Published:** 2015-05-19

**Authors:** Susan Garthus-Niegel, Matthias Nübling, Stephan Letzel, Janice Hegewald, Mandy Wagner, Philipp S. Wild, Maria Blettner, Isabella Zwiener, Ute Latza, Sylvia Jankowiak, Falk Liebers, Andreas Seidler

**Affiliations:** Medical Faculty Carl Gustav Carus, Institute and Polyclinic of Occupational and Social Medicine, TU Dresden, Fetscherstr. 74, 01307 Dresden, Germany; Department of Psychosomatics and Health Behavior, Norwegian Institute of Public Health, Oslo, Norway; FFAS, Freiburg Research Center for Occupational and Social Medicine, Bertoldstr. 27, 79098 Freiburg, Germany; Institute of Occupational, Social and Environmental Medicine, University Medical Center of the Johannes Gutenberg University Mainz, Obere Zahlbacher Straße 67, 55131 Mainz, Germany; Center for Thrombosis and Hemostasis, University Medical Center Mainz, Langenbeckstr. 1, 55131 Mainz, Germany; Department of Medicine 2, University Medical Center Mainz, Langenbeckstr. 1, 55131 Mainz, Germany; German Center for Cardiovascular Research (DZHK), University Medical Center Mainz, Langenbeckstr. 1, 55131 Mainz, Germany; Institute of Medical Biostatistics, Epidemiology and Informatics (IMBEI), University Medical Center, Johannes Gutenberg University Mainz, Obere Zahlbacher Straße 69, 55131 Mainz, Germany; Federal Institute for Occupational Safety and Health (BAuA), Nöldnerstr. 40-42, 10317 Berlin, Germany

**Keywords:** Mobbing, Gutenberg Health Study, COPSOQ, Stress and strain, Psychometric evaluation

## Abstract

**Purpose:**

Despite its highly detrimental potential, most standard questionnaires assessing psychosocial stress at work do not include mobbing as a risk factor. In the German standard version of COPSOQ, mobbing is assessed with a single item. In the Gutenberg Health Study, this version was used together with a newly developed short scale based on the Leymann Inventory of Psychological Terror. The purpose of the present study was to evaluate the psychometric properties of these two measures, to compare them and to test their differential impact on relevant outcome parameters.

**Methods:**

This analysis is based on a population-based sample of 1441 employees participating in the Gutenberg Health Study. Exploratory and confirmatory factor analyses and reliability analyses were used to assess the mobbing scale. To determine their predictive validities, multiple linear regression analyses with six outcome parameters and log-binomial regression models for two of the outcome aspects were run.

**Results:**

Factor analyses of the five-item scale confirmed a one-factor solution, reliability was *α* = 0.65. Both the single-item and the five-item scales were associated with all six outcome scales. Effect sizes were similar for both mobbing measures.

**Conclusion:**

Mobbing is an important risk factor for health-related outcomes. For the purpose of psychosocial risk assessment in the workplace, both the single-item and the five-item constructs were psychometrically appropriate. Associations with outcomes were about equivalent. However, the single item has the advantage of parsimony, whereas the five-item construct depicts several distinct forms of mobbing.

## Background

Mobbing, as a pertinent psychosocial stress factor at work, has generated considerable research interest over the last two decades (Kivimäki et al. 2003; Rodríguez-Carballeira et al. 2010). Mobbing is not merely an interpersonal issue, but it is an organizational dynamic that affects all who are exposed, including witnessing colleagues and the workplace as a whole (Hoel et al. 2003; Mayhew and Chappell 2007; Salin 2003). Various causes of mobbing have been identified: organizational causes, individual characteristics of both the mobbing targets and bullies, as well as workgroup characteristics such as low workgroup identification (Escartín et al. 2013). As a consequence, recent studies have shown that mobbing is one of the factors most closely related to health-related outcomes such as general health and burnout (Nübling et al. 2010, 2013). It has even been suggested that being bullied at work is more distressing for employees than all other work-related stress factors put together (Einarsen et al. 2009; Einarsen and Mikkelsen 2003). Einarsen et al. (2003) define mobbing as follows: “Bullying at work means harassing, offending, socially excluding someone or negatively affecting someone’s work tasks. In order for the label bullying (or mobbing, both terms are used interchangeably) to be applied to a particular activity, interaction, or process, it has to occur repeatedly and regularly (e.g., weekly) and over a period of time (e.g., about 6 months).”

In a recent study (Rodríguez-Carballeira et al. 2010), a taxonomy of mobbing strategies has been suggested, containing six principal categories of mobbing with various subcategories. The first three categories refer to strategies related to indirect aggressive behaviors (delivery of harm through the actions of other agents or through assaults on persons or objects valued by the victim). The last three categories relate to direct aggressive behaviors (harm is delivered directly to the victim) and the experiences of the individuals affected (Baron and Neuman 1996). The categories relating to direct aggressive behaviors corresponded with the highest degree of severity (Rodríguez-Carballeira et al. 2010).

Despite its highly detrimental potential, widely used questionnaires assessing psychosocial stress at work such as the effort–reward imbalance (ERI) Questionnaire and the Copenhagen Psychosocial Questionnaire (COPSOQ, version I) do not include mobbing as a risk factor (Kristensen et al. 2005; Siegrist 1996, 2001). In the German standard version of COPSOQ, the subject is covered with a single item (Nübling et al. 2006) taken from the German BIBB/IAB survey (Zentralarchiv für Empirische Sozialforschung Köln 1999). Yet, single-item measures have been criticized because of reliability and validity concerns. Also, internal consistency and error variance in latent variable modelling cannot be estimated (Bollen 1989). If a construct, such as “overall job satisfaction,” is sufficiently narrow and unambiguous, it may be feasible to administer a single-item scale (Wanous et al. 1997). Mobbing however comprises a variety of different strategies with differing degrees of severity (Rodríguez-Carballeira et al. 2010)—thus, a multi-item assessment might be more appropriate.

Recently, Escartin et al. (2010) developed a new mobbing scale that covers the most severe mobbing strategies. However, this scale contains 12 items, which may be too many to incorporate in an already exhaustive questionnaire as the COPSOQ addressing the whole range of psychosocial factors. Therefore, in the Gutenberg Health Study (GHS), we set out to compare a new five-item scale whose items were derived from the Mobbing Scale “Leymann Inventory of Psychological Terror” (LIPT31; Leymann 1996), with the single item included in the COPSOQ. In order to generalize and to examine whether the results of the two measurements (the five-item scale and the single-item scale) were congruent not only overall but also for subgroups, we also set out to perform subgroup analyses according to gender, age, and profession.

### Aims of the present study

The aims of the present study were (1) to examine whether the proposed five-item scale adequately represents the construct “mobbing” and to test its psychometric properties; (2) to compare the proposed five-item scale with the single item that measures mobbing in the German version of the COPSOQ, both overall and in subgroup analyses; and (3) to test the differential impact of the measured mobbing strategies on relevant outcome parameters.

## Methods

### Design and participants

The GHS is designed as a population-based, prospective, observational, single-center cohort study in the Rhine-Main region in Western Germany (Beutel et al. 2012; Wild et al. 2010). The primary aim was to evaluate and improve cardiovascular risk stratification. The sample was drawn randomly from the governmental local registry offices in the city of Mainz and the district of Mainz-Bingen. The sample was stratified 1:1 for sex and residence (urban and rural) and in equal strata for decades of age. Individuals between 35 and 74 years of age were enrolled, and written informed consent was obtained from all participants. Exclusion criteria were insufficient knowledge of the German language and physical or psychological inability to participate in the examinations at the study center. The study protocol and sampling design were approved by the local ethics committee and by the local and federal data safety commissioners.

In the period between April 2007 and October 2008, the first 5000 subjects were enrolled in the GHS, and 2783 (56 %) participants were employed at the time of enrollment. About half of the participating employees (*N* = 1441, 52 %) completed the COPSOQ questionnaire. The other half completed an alternative questionnaire (the ERI questionnaire). More details about these procedures have been reported previously (Nübling et al. 2013).

In the present study, we investigated the baseline (cross-sectional) data of all 1441 cases that had completed the COPSOQ questionnaire (only persons with employment were asked to fill out the questionnaire). The mean age of the participants was 49.1 years (standard deviation (SD) = 7.8), and distribution of grouped age was as follows: 35–44 years: 30.9 %, 45–54 years: 43.1 %, 55–64 years: 24.1 %, 65–74 years: 1.9 %. There were fewer females (46.2 %) in the sample, as fewer women were employed than men (51.8 and 59.5 %, respectively).

### Measures

The German standard version of the COPSOQ used in this study (Nübling et al. 2006) consists of five thematic domains including 25 constructs. The first four thematic domains represent the psychosocial factors at work: “demands” (four scales), “influence and development” (five scales), “interpersonal relations and leadership” (nine scales), and “further parameters” (one scale on insecurity at work in the present study). The fifth domain represents “strain” (six constructs), assessing the reactions of the employees on the workplace situation as the internal outcome parameters. The six outcome factors are “job satisfaction” (seven items, *α* = 0.83), “intention to leave” (single item), “general health” (single item), “burnout” (six items, *α* = 0.89), “cognitive stress” (four items, *α* = 0.84), and “satisfaction with life” (five items, *α* = 0.90). According to the validation study (Nübling et al. 2006), mobbing is one of the nine constructs in the domain “interpersonal relations and leadership.” As mentioned above, it is measured with a single item in the German version of the COPSOQ: “How often do you feel unjustly criticized, bullied or shown up in front of others by your colleagues and your superior?” (The original questions in German are given in “Appendix”). Answer categories were “always,” “often,” “sometimes,” “seldom,” and “never/hardly ever.”

Parallel to the single item in the COPSOQ, we assessed mobbing with a new five-item scale. This scale was developed in a pilot study (*N* = 223) conducted among employees of a regional state authority. Items were derived from the well-established and previously validated “Leymann Inventory of Psychological Terror” (LIPT31; Leymann 1996). We aimed at developing an economic scale with maximum sensitivity. Therefore, the strategy to pick items was as follows: Whenever a participant qualified as a mobbing victim by ticking off at least one of the 31 items of the LIPT31 with “at least one time per week for at least 6 months,” one of the items of the new short scale should be among these items. This strategy resulted in a minimum number of items (five) that would cover the breadth of the construct sufficiently. The resulting items were (1) “Do you get intentionally interrupted during oral contributions?” (2) “Does it happen that you receive no response/reaction, when you want to speak to someone?” (3) “Do you get blamed for others’ mistakes or general operational problems?” (4) “Were important influential or working areas taken away from you?” (5) “Did you receive unpleasant sexual offers or did you get sexually harassed?” If an item was endorsed with “yes,” the respondents were asked about the frequency: “daily,” “at least one time per week,” “at least one time per month,” “less than one time per month.” The five-item mobbing scale was then tested in a larger second study (*N* = 3292), involving two different regional state authorities, where the five-item scale correlated (organization A: *r* = 0.735, *N* = 666; organization B: *r* = 0.716, *N* = 2626) with the LIPT31 index (Eisermann and de Costanzo 2011).

In accordance with the standard procedure for the COPSOQ and in order to compare the results of the single-item and the five-item scales, the answer categories in the GHS were presented as “daily/almost daily,” “at least one time per week,” “at least one time per month,” “less than one time per month,” and “never,” and all answer categories were transformed to values between 0 and 100. The scale value of the five-item mobbing construct was calculated as the mean value of the single items if at least three items were answered (if two or less items were answered, no scale value was calculated—it was set to missing).

### Statistical analysis

Descriptive analyses and ANOVA were carried out in order to obtain means and standard deviations of the mobbing constructs and their items and to check for statistical differences (*p* < 0.05). Occupations were manually double-coded according to the classification of occupations of the Federal Statistical Office, Germany (KldB 2010, Klassifikation der Berufe; Bundesagentur für Arbeit 2011). In the analyses related to occupations, we included only professional groups represented by at least 20 employees. For all other analyses, all cases available were included.

In order to check the psychometric properties of the five-item scale, we carried out a reliability analysis, a principal component analysis (SPSS version 18: command “factor analysis,” mean substitution of missings, varimax rotation), and a confirmatory factor analysis (Stata version 13). Further, to determine the association with outcome factors of the five-item scale and to compare it with the single-item scale, we carried out multiple linear regression analyses with all six outcome scales and ran log-binomial regression models for the outcome scales “general health” and “intention to leave.” “General health” was chosen, as it has been proven to be an important (negative) health-related outcome of mobbing (Nübling et al. 2013), and it is a broad outcome that may include various harmful health-related outcomes. Also, Hasselhorn, Tackenberg, and Müller demonstrate in the NEXT study (nurses early exit study) that “intention to leave” is also a pertinent outcome of work stress (Hasselhorn et al. 2003). As a marker for severe dissatisfaction at work, “intention to leave” was therefore used as another outcome for the log-binomial regression analyses.

For these analyses, the mobbing constructs were dichotomized in “less than one time per week” and “at least one time per week” for the five items and “sometimes, often, and always” versus “seldom and never” for the single item. The outcome scales “general health” and “intention to leave” were ranging from 0 to 100. The cutoff for the log-binomial regression was set at ≥50. The models were run for both the entire group and females and males separately. Prevalence risk ratios were calculated with SAS as suggested by Spiegelman and Hertzmark (Spiegelman and Hertzmark 2005).

## Results

Cronbach’s alpha for the five-item scale was 0.65. The principal component analysis of the scale resulted in a one-factor solution with 41.89 % explained variance. Factor loadings were as follows: “intentionally interrupted” 0.80, “received no response/reaction” 0.77, “blamed for others’ mistakes” 0.70, “important working areas taken away” 0.55, and “sexual harassment” 0.29. The confirmatory factor analysis of the five-item scale confirmed the adequacy of the five items to measure mobbing as it resulted in a latent factor with a good fit [root mean square error of approximation (RMSEA) = 0.06, standardized root mean square residual (SRMR) = 0.03, comparative fit index (CFI) = 0.97, Tucker–Lewis index (TLI) = 0.95. The Chi-squared test for the difference between observed and expected covariance matrices was significant [*χ*^2^ = 26.79, *df* = 5, *χ*^2^/*df* = 5.36, (*p* < 0.001)]; however, in case of large sample sizes, this test is nearly always significant.

### Comparison of mobbing constructs

Overall, numerically slightly higher scores on a scale ranging from 0 to 100 were obtained with the single-item scale compared to the five-item scale. With regard to gender, no statistical differences were found for the single-item scale. However, in the five-item scale, males had higher values (scale mean of 13.6 points in males vs. 10.9 points in females). The biggest differences on the level of items were found for “blamed for others’ mistakes” (males: 21.4, females 14.4); the values in males were not higher for the last two items (“important areas taken away” and “sexual harassment”). Tables 1 and 2 show the distribution of the two mobbing scales for different age groups and occupations, respectively. With regard to different age groups, no statistical differences were found, neither for the single-item nor the five-item scale. However, the age group of 65–74 years had elevated values for both the single-item scale (mean value of 22.7 vs. overall mean of 15.2) and the item “intentionally interrupted” in the five-item scale (mean value of 31.3 vs. overall mean of 21.7). Probably due to the low number of employed people in that age group (*N*_male_ = 6 and *N*_female_ = 6), a statistical difference could not be shown. Regarding occupational groups, we ranked the occupations by the results of the single-item scale (Table 2). “Sales personnel” topped the list with the highest mobbing score (mean = 20.4), whereas “teachers” reported the lowest scores (mean = 7.3). Generally, similar tendencies could be observed for the five-item scale and the single item. Nevertheless, there were also discrepancies, e.g., even though “teachers” had the lowest mobbing score on the single-item scale (mean = 7.3), they ranked fourth (mean = 14.5) on the five-item scale (Table 2).

**Table 1:** Mean and standard deviation of mobbing scales (range from min. 0 to max. 100) for different age groups

	Age in years			
	35–44 (*N* = 400)^a^	45–54 (*N* = 544)	55–64 (*N* = 278)	65–74 (*N* = 12)
	Mean (SD)			
Single-item scale	16.0 (20.6)	15.0 (19.0)	14.0 (19.4)	22.7 (28.4)
Five-item scale	12.1 (13.7)	13.2 (13.6)	10.9 (12.9)	12.9 (16.8)
(1) Intentionally interrupted	21.0 (27.1)	23.6 (28.1)	18.8 (27.2)	31.3 (37.1)
(2) Received no response/reaction	14.1 (22.3)	14.0 (22.6)	12.0 (20.2)	16.7 (28.9)
(3) Blamed for others’ mistakes	17.9 (23.1)	19.4 (24.8)	15.9 (24.0)	12.5 (22.6)
(4) Important working areas taken away	6.3 (15.7)	8.3 (17.9)	7.5 (19.0)	4.2 (9.7)
(5) Sexual harassment	0.9 (5.4)	0.9 (7.0)	0.7 (4.2)	0.0 (0.0)

^a^Due to missing values on some of the items, *N* varied between 400 and 398, 544 and 539, 278 and 275, respectively. The group 65–74 years of age had *N* = 12 on all items/scales

**Table 2:** Mean and standard deviation of mobbing scales (range from min 0 to max 100) for different occupational groups

Occupational group	Mean (SD)		Number of subjects
	Single-item scale^a^	Five-item scale	
Sales personnel	20.4 (20.8)	17.1 (11.9)	27
Technicians	18.6 (22.3)	15.9 (15.6)	33 and 31
Social occupations	18.4 (20.3)	13.7 (14.3)	52 and 53
Occupations of land transport	18.2 (22.6)	11.1 (14.3)	37
Other occupations in the health sector	17.5 (21.3)	13.4 (14.0)	68 and 67
Wholesale and retail salesmen, purchasing, and sales professionals	17.3 (25.3)	15.0 (18.2)	26
Workforces without further specification	14.9 (21.0)	9.4 (14.4)	80 and 79
Accounting clerks, computer scientists	14.4 (18.5)	12.7 (13.1)	85 and 87
Office jobs, commercial employees	13.3 (17.6)	10.4 (12.3)	165
Assemblyman, representatives	12.9 (19.9)	11.9 (13.0)	34 and 33
Occupations in management, management consultancy, and company audit	12.2 (16.8)	12.0 (12.0)	121
Banking and insurance professionals	11.7 (17.4)	9.1 (9.7)	45
Engineers	11.1 (16.6)	10.5 (10.2)	43
Teachers	7.3 (15.3)	14.5 (14.9)	61 and 62

^a^Occupations were ranked by the results of the single-item scale

### Differential impact on outcome parameters

Both the single-item scale and the five-item scale were associated with all six outcome scales, i.e., being bullied was related to increased strain at work in terms of less satisfaction and lower health-related outcomes. Effect sizes were similar for both scales (Table 3).

**Table 3:** Multiple linear regression analyses with all six outcome scales

	Single-item scale		Five-item scale	
	*β* (SE)	*R* ^2^	*β* (SE)	*R* ^2^
Job satisfaction	−0.36*** (0.02)	0.14	−0.32*** (0.03)	0.11
Intention to leave	0.24*** (0.03)	0.06	0.28*** (0.04)	0.09
General health	−0.17*** (0.02)	0.04	−0.22*** (0.04)	0.06
Burnout	0.27*** (0.02)	0.12	0.31*** (0.03)	0.14
Cognitive stress	0.22*** (0.03)	0.06	0.27*** (0.04)	0.09
Satisfaction with life	−0.18*** (0.03)	0.04	−0.19*** (0.04)	0.04

*** *p* < 0.001

The results of the log-binomial regression analyses confirmed that being exposed to mobbing, as it was measured in this study, was associated with an increased overall risk of strain at work. The overall prevalence risk ratios for both “intention to leave” and poor “general health” were somewhat higher for the single-item scale than for the five-item scale (Table 4). However, on the level of individual items, for “intention to leave,” item four of the five-item scale (“important working areas taken away”) yielded a little higher prevalence risk ratio than did the single-item scale (PR of 2.3 vs. 2.0, respectively; Table 4). In addition, for “general health,” item 2 (“received no response/reaction”) yielded a somewhat higher prevalence risk ratio (PR of 3.3 vs. 3.0 for item 2 and single-item scale, respectively).

**Table 4:** Prevalence risk ratios (PR) for mobbing items and the outcome scales “intention to leave” and “general health”

	“Intention to leave”						“General health”					
Variable	Females (*n* = 637)		Males (*n* = 753)		All (*n* = 1390)		Females (*n* = 644)		Males (*n* = 754)		All (*n* = 1398)	
	*N* (%)	Adj. PR^a^ (95 % CI)	*N* (%)	Adj. PR^a^ (95 % CI)	*N* (%)	Adj. PR^b^ (95 % CI)	*N* (%)	Adj. PR^a^ (95 % CI)	*N* (%)	Adj. PR^a^ (95 % CI)	*N* (%)	Adj. PR^b^ (95 % CI)
Single-item scale												
Seldom and never (0)	199 (35.3)	1.0 (–)	215 (34.7)	1.0 (–)	414 (35.0)	1.0 (–)	44 (8.7)	1.0 (–)	43 (6.9)	1.0 (–)	87 (7.3)	1.0 (–)
Sometimes, often, and always (1)	12 (92.3)	2.6 (2.0–3.4)	6 (46.2)	1.4 (0.8–2.5)	18 (69.2)	2.0 (1.6–2.6)	4 (30.8)	4.0 (1.7–9.2)	2 (15.4)	2.0 (0.5–7.1)	6 (23.1)	3.0 (1.5–6.2)
Five-item scale												
No/<1× per week (0)	158 (33.1)	1.0 (–)	154 (31.2)	1.0 (–)	312 (32.1)	1.0 (–)	35 (7.3)	1.0 (–)	28 (5.7)	1.0 (–)	63 (6.5)	1.0 (–)
≥1× per week (1)	55 (56.7)	1.7 (1.4–2.1)	68 (48.6)	1.5 (1.2–1.9)	123 (51.9)	1.6 (1.4–1.9)	12 (12.4)	1.6 (0.9–3.0)	18 (12.9)	2.3 (1.3–4.0)	30 (12.7)	1.9 (1.3–2.8)
(*1*) *intentionally interrupted*												
No/<1× per week (0)	183 (35.6)	1.0 (–)	186 (33.5)	1.0 –	369 (34.5)	1.0 (–)	38 (7.4)	1.0 (–)	34 (6.1)	1.0 (–)	72 (6.7)	1.0 (–)
≥1× per week (1)	32 (50.0)	1.4 (1.1–1.9)	37 (44.6)	1.3 (1.0–1.7)	69 (46.9)	1.4 (1.1–1.7)	9 (13.8)	1.8 (0.9–3.5)	12 (14.5)	2.4 (1.3–4.4)	21 (14.2)	2.1 (1.2–3.2)
(*2*) *received no response/reaction*												
No/<1× per week (0)	197 (36.1)	1.0 (–)	203 (34.0)	1.0 (–)	400 (35.0)	1.0 (–)	40 (7.3)	1.0 (–)	38 (6.4)	1.0 (–)	78 (6.8)	1.0 (–)
≥1× per week (1)	18 (58.1)	1.6 (1.2–2.2)	20 (50.0)	1.4 (1.0–1.9)	38 (53.5)	1.5 (1.2–1.9)	7 (22.6)	3.1 (1.5–6.4)	8 (20.5)	3.4 (1.7–6.6)	15 (21.4)	3.3 (2.0–5.3)
(*3*) *blamed for others’ mistakes*												
No/<1× per week (0)	198 (35.7)	1.0 –	187 (32.5)	1.0 (–)	385 (34.1)	1.0 (–)	43 (7.7)	1.0 (–)	38 (6.6)	1.0 (–)	81 (7.2)	1.0 (–)
≥1× per week (1)	16 (61.5)	1.8 (1.3–2.4)	35 (56.5)	1.6 (1.3–2.1)	51 (58.0)	1.7 (1.4–2.1)	4 (14.8)	1.8 (0.7–4.7)	8 (12.9)	2.1 (1.0–4.2)	12 (13.5)	2.0 (1.1–3.4)
(*4*) *important working areas taken away*												
No/<1× per week (0)	199 (35.5)	1.0 (–)	214 (34.2)	1.0 (–)	413 (34.8)	1.0 (–)	44 (7.8)	1.0 (–)	42 (6.7)	1.0 (–)	86 (7.2)	1.0 (–)
≥1× per week (1)	14 (82.4)	2.3 (1.8–3.0)^c^	8 (66.7)	1.9 (1.3–2.8)	22 (75.9)	2.3 (1.9–2.7)	3 (16.7)	1.9 (0.6–5.5)	4 (33.3)	4.4 (1.9–10)	7 (23.3)	2.7 (1.4–5.4)
(*5*) *sexual harassment*												
No/<1× per week (0)	213 (36.8)		223 (34.9)		436 (35.8)		45 (7.8)		46 (7.2)		91 (7.5)	
≥1× per week (1)	1 (100)		–		1 (100)		1 (50)		–		1 (50)	

Missing values were analyzed as a separate category (data not shown)

^a^Adjusted for age, ^b^ adjusted for age and gender, ^c^ crude PR (model did not converge when adjusted for age)

We also found gender differences. According to the single-item scale, bullied females had a noticeably higher risk of an intention to quit their job (PR of 2.6 vs. 1.4, respectively). However, according to the five-item scale, the risk of an intention to leave one’s job was similar for bullied females and males (PR of 1.7 vs. 1.5, respectively) (Table 4). Also for poor “general health,” the single-item scale yielded higher prevalence risk ratios for bullied females (PR of 4.0 vs. 2.0, respectively). On the contrary, according to the five-item scale, the prevalence risk ratios were lower for bullied females (PR of 1.6 vs. 2.3, respectively). This difference was particularly pronounced for item 4, “important working areas taken away” (PR of 1.9 vs. 4.4 for females and males, respectively).

## Discussion

This population-based study aimed at (1) examining the adequacy of the proposed five-item scale; (2) comparing the proposed five-item scale with the single item that measures mobbing in the German standard version of the COPSOQ; and (3) testing the differential impact of the measured mobbing strategies on relevant outcome parameters.

### Five-item scale

Inter-item correlation of the proposed five-item scale was not particularly high, and Cronbach’s alpha was below 0.70, which has been suggested as a cutoff for acceptable internal consistency reliability (Nunnally and Bernstein 1994). However, as mobbing comprises a variety of different strategies, our emphasis laid on developing a scale with high sensitivity that covers the breadth of the construct sufficiently rather than maximizing internal consistency. Item collections intended to reflect a broad construct such as mobbing will, on average, correlate less highly with each other than will items reflecting a narrow, more tightly defined construct, because each item can only represent a smaller portion of the broad construct (Smith et al. 2000). “Sexual harassment” had the lowest factor loading (0.29). Indeed, regarding sexual harassment, some researchers assume it to be conceptually different from other mobbing strategies (Bergman and Henning 2008; Kauppinen and Tuomola 2008). However, already in 1976, the American occupational psychiatrist Brodsky considered sexual harassment as only one of five types of work harassment (Brodsky 1976), and also the Scandinavian concept of mobbing clearly includes “sexual harassment” (Einarsen 2000; Einarsen and Skogstad 1996). Consequently, the Swedish Heinz Leymann included sexual harassment in his mobbing inventory, the LIPT31, which represents the basis for the newly developed five-item scale (Leymann 1996). Even the WHO, which recognizes Leymann as the first researcher who dealt scientifically with the issue of “mobbing,” includes sexual harassment in their definition of mobbing (Cassitto et al. 2003). Our empirical findings support this notion, because both the principal component analysis and the confirmatory factor analysis attested the adequacy of the five-item scale as a one-factor mobbing measure, i.e., despite the somewhat lower factor loading sexual harassment belonged to the construct.

### Comparison of mobbing scales and differential impact

The single-item scale yielded slightly higher numerical scores for mobbing compared to the five-item scale (15.2 points vs. 12.3 points on a 0–100 scale). In particular, two items of the five-item scale resulted in low scores: item 4 “important working areas taken away” (7.4 points) and item 5 “sexual harassment” (0.9 points). Concerning the latter, research has demonstrated that while sexual harassment at the workplace is severe and linked to a wide range of negative outcomes in victims (Berdahl and Raver 2011; Cortina and Berdahl 2008), sexual harassment is not as common as nonsexual mobbing strategies with men being considerably less frequently affected than women (Kauppinen and Tuomola 2008). This explains the lower scores for sexual harassment in our study and why we were not able to estimate prevalence risk ratios for men with regard to this item. Concerning item 4, a low score is not surprising either, as an incidence of “at least one time per week” for at least 6 months was required to be defined as mobbing (Leymann 1996). Not many employees have such a high number of working areas that one can be taken away every week. At the same time, once a work area is taken, it may have significant long-term detrimental effects.

The items of the proposed five-item scale fit well with the taxonomy suggested by Rodríguez-Carballeira et al. (2010). Items 1 “intentionally interrupted”, 2 “received no response/reaction,” and 5 “unpleasant sexual offers/sexual harassment” would belong to the subcategory “emotional abuse.” Item 3 “blamed for others’ mistakes” would belong to the subcategory of “professional discredit and denigration,” and item 4 “important working areas taken away” would be categorized as “devaluation of the role in the workplace.” Thus, the five items of the proposed scale cover all three subcategories of the *direct mobbing strategies* and thereby measure the most important and severe mobbing strategies (Rodríguez-Carballeira et al. 2010). In support of this, the five-item scale was related to all six outcome measures, predicting unfavorable consequences of being bullied (Tables 3, 4). Compared to the EAPA-T scale (Escartin et al. 2010), our new scale covered three of four categories in the EAPA-T (all except “control and manipulation of the work context.”. With such a short scale, it was not possible to cover as many categories. However, while this fourth category belongs to the less harmful indirect mobbing strategies (Escartin et al. 2010; Rodríguez-Carballeira et al. 2010), due to their severity, we deem the direct strategies as most relevant.

Also, the single-item scale “How often do you feel unjustly criticized, bullied or shown up in front of others by your colleagues and your superior?” seems to measure *direct* psychological abuse according to the categorization of Rodríguez-Carballeira et al. (2010). The scale was also related to all six outcome measures, and effect sizes were comparable to those of the five-item scale. However, according to the log-binomial regression analyses, negative outcomes such as “intention to leave” and “general health” were somewhat better predicted by the single-item scale. The reason for this may be the open wording of the item without pinpointing concrete acts, which allows for measuring a wide array of mobbing strategies of *direct psychological abuse*.

On the other hand, the differential impact of mobbing became evident when the five-item scale was applied. We could demonstrate that health risks may differ depending on the particular mobbing strategy. This finding is in line with the Rodríguez-Carballeira study that suggests that mobbing strategies vary in their severity (Rodríguez-Carballeira et al. 2010). In addition, the risks differed depending on the outcome parameters that were measured (in the present study “intention to leave” and “general health”).

We also found gender-specific differences. Mobbing as measured with the single item appears to affect females to a larger degree (relation to “intention to leave” and “general health”). This finding is supported by a recent study that examined the “gendered nature of perceptions of mobbing” (Escartin et al. 2011). In this study, females rated the severity of many types of mobbing as more severe than males did. Applying the five-item scale however, mobbing was not associated with a higher risk of an “intention to leave” for females compared to males. The risk of poor “general health” was even higher for males, according to the five-item scale. Males seemed to be particularly affected if “important working areas (were) taken away” from them. It is conceivable that men are more affected by work-related forms of mobbing (Escartin et al. 2011). In support of this, Escartin et al. (2011) found that men emphasized abusive working conditions and (to a certain degree) devaluation of their professional role more than women in their definitions of mobbing.

Discussing causality and the detrimental impact of mobbing at work, we cannot rule out a common source bias, as the results are based on self-report only. In a similar vein, the cross-sectional nature of this study and the potential reverse causality has to be kept in mind. For instance, it has been shown that workplace mobbing may impair the worker’s health status (Hansen et al. 2006; Niedhammer et al. 2008). At the same time, it is conceivable that a deteriorated health status may result in “important working areas taken away” (item 4). Currently, the GHS is assessing 5-year follow-up data. These data will give the opportunity to control for initial states and to prospectively analyze the impact of mobbing on work-related outcomes and even hard outcomes such as cardiovascular events.

## Conclusion

In this paper, two constructs assessing mobbing were examined. Within a population-based cross-sectional study, both scales proved to be psychometrically appropriate as both were markedly and very similarly associated with relevant self-reported outcome parameters. The proposed five-item scale could even demonstrate a differential impact on the various outcomes. Taking the LIPT31 as a gold standard, the five-item scale also showed a very high sensitivity. However, because of the broadness of the construct, the resulting Cronbach’s alpha was low. Particularly, items 4 and 5 were somewhat problematic. Multiple-item measures are normally to be preferred because of reliability concerns (Nunnally 1978). Nonetheless, if a single-item measure demonstrates predictive validity (in terms of correlation or regression coefficients) equal to that of the multiple-item measure, reliability becomes a minor issue (Bergkvist and Rossiter 2007). Therefore, until the five-item scale is further improved, the parsimonious single-item measure in the COPSOQ seems to be a good alternative to measure mobbing at work.

The present study has a cross-sectional design; hence, its results have to be interpreted cautiously. Currently, the GHS is assessing the 5-year follow-up data. Further conclusions about the relationship between mobbing or certain mobbing strategies and defined illnesses, such as cardiovascular events, will be possible when these data are available.

## Appendix: Wording of questions in German questionnaire

1. Single-item mobbing:
  “Wie oft fühlen Sie sich durch Kollegen und Vorgesetzte zu unrecht kritisiert, schikaniert oder vor anderen bloßgestellt?” (answer categories: immer, oft, manchmal, selten, nie/fast nie)
2. Five items mobbing:
  1. Werden Sie bei mündlichen Ausführungen/Beiträgen absichtlich unterbrochen?
  2. Kommt es vor, dass auf Sie nicht reagiert wird, wenn Sie jemanden ansprechen wollen? (Hiermit ist aber nicht gemeint, dass Sie von Jemandem nicht zurückgegrüßt wurden.)
  3. Werden Sie für Fehler der anderen oder für allgemeine betriebliche Probleme verantwortlich gemacht?
  4. Sind Ihnen wichtige Einfluss- und Tätigkeitsbereiche weggenommen worden?
  5. Sind Ihnen unangenehme verbale sexuelle Angebote gemacht worden oder sind Sie sexuell belästigt worden?
    (answer categories: täglich/fast täglich, mind. 1 mal pro Woche, mind. 1 mal im Monat, seltener als 1 mal pro Monat, nie)
